# A Real-World Study of Prognosis of N0M0 Hepatocellular Carcinoma with Hepatic Resection Based on SEER Database

**DOI:** 10.1155/2020/2357840

**Published:** 2020-04-01

**Authors:** Guangxi Zhu, Wensheng Wang, Qin Liu, Dongfeng Chen, Liangzhi Wen

**Affiliations:** Department of Gastroenterology, Daping Hospital, Army Medical University, Chongqing, China

## Abstract

**Aim:**

To develop and validate a simple-to-use nomogram for prediction of 3-/5-year survival in patients with N0M0 hepatocellular carcinoma after curative liver resection. *Patients and Methods*. Patients diagnosed HCC with hepatic resection in the Surveillance, Epidemiology, and End Results (SEER) database were included to identify prognostic factors of overall survival. Multivariate Cox regression were used to create a nomogram.

**Results:**

We identified 4856 HCC with hepatic resection from the SEER database. A nomogram to predict long-term survival with a C-index 0.667 (95% CI, 0.653 to 0.681) is more efficient than TNM staging with a lower C-index 0.613 (95% CI, 0.597 to 0.629). The C-index was confirmed to be 0.663 (95% CI, 0.640 to 0.686) through validation, suggesting a good discrimination and a good prediction capability.

**Conclusions:**

The nomogram is a simple and effective screening tool for assessing the prognosis of HCC with hepatic resection and assists with the planning of individual postoperative surveillance protocols.

## 1. Introduction

Hepatocellular carcinoma (HCC) is one of the most common and most malignant tumors, with seventh incidence rate and second cancer-related mortality rate worldwide [1], while both rates are ranked third in China [2]. To date, surgical treatment is the best treatment for HCC patients without regional lymph node metastasis and distant metastasis, and hepatic resection remains the best therapeutic option for their potential curative outcomes, which can significantly prolong their survival. But around 50% of these patients suffered relapse within 2 years after surgery. Let alone a large number of patients have already lost their chance of surgery at the time of diagnosis [3]. However, there are currently not many reports on the effects of hepatic resection, and also a lack of effective methods for assessing the patients' prognosis. In general, TNM staging is strongly correlated with survival, and the American Joint Committee on Cancer (AJCC)just released its 8th edition [4]. Nevertheless, different prognosis could still be observed at the same stage according to TNM staging system. The difference may be due to other prognostic factors such as age, sex, etc. Therefore, a more refined method for predicting individualized survival of HCC is in needed, and a nomogram is such a good method to serve this purpose. Nomograms have been designed to serve in many different human cancers, and they have all shown a good advantage over other traditional staging systems [5–7]. The Surveillance, Epidemiology, and End Results (SEER) database is an important resource that based on American people for understanding the impact of pathological diagnosis on population groups [8]. The purpose of our study was to perform a Nomogram analysis and validation of the prognosis of surgically treated HCC patients based on the SEER database.

## 2. Patients and Methods

### 2.1. Data Source and Study Cohort

We visited the SEER database (https://seer.cancer.gov/) and used SEERStat8.3.5 software to obtain online information on hepatocellular carcinoma patients (from 1973 to 2015) and related prognostic information (site recode ICD-O-3/WHO 2008 = Liver). A total of 126024 cases were viewed, and 4856 met the inclusion criteria: (1) patients with clear histopathological diagnosis (confirmed as positive histology); (2) patients with tissue differentiation grade I, II, III, or IV based on ICD-O-2 criteria; and (3) no regional lymph node metastasis or extrahepatic metastasis (N0M0). We enrolled these 4856 patients in this present study, including 2254 patients who died at the end of the follow-up. The mean follow-up of these 4856 HCC patients is 38 months.

### 2.2. Nomogram Construction and Validation

For nomogram construction and validation, we randomly assigned 70% of the patients to the derivation cohort (*n* = 3400) and 30% to validation cohort (*n* = 1456). Cox proportional hazard models were used to assess the univariate and multivariate analyses of the risk factors associated with patient survival. The construction of survival nomogram was based on the multivariate analysis. Hazard ratios are presented with 95% CI. The nomogram was validated using the concordance index (C-index) and calibration plots. The C-index measures the probability of concordance between predicted and observed survival, similar to the area under the receiver operating characteristic (ROC) curve for censored data. A calibration plot was used to assess the prediction accuracy of the nomogram by plotting the actual survival against the nomogram-predicted survival probabilities. The AJCC system, as one of the important risk factors, was specially pointed out in this study for it has been widely used; we compared the three editions (6th, 7th, and 8th) of AJCC system and proved that the 8th version overweighed the others, so we chose 8th AJCC to compare with our nomogram for their effectiveness on predicting the prognosis of HCC patients. As no personal confirmation information was contained in this present study, and all the datasets used were public and available online, so personal informed consent and ethical approval are not required.

### 2.3. Statistical Analysis

Variables used in this study include vital status, survival months, age, race, sex, tumor differentiation grade, tumor size, AJCC staging system, surgery of primary site, radiation, chemotherapy, level of *α*-fetoprotein prior to treatment, AJCC classifies fibrosis scores (also called Ishak score), and marital status. Excel 2007 was used to organize the data. The statistical analyses were performed using R software version 3.5.2 (https://www.r-project.org) with the survival and design packages (rms, caret, ROCR, rmda, and survivalROC). Overall survival (OS) was calculated from diagnosis to death from any cause. The Kaplan-Meier method and log-rank test were used to evaluate OS differences. The significant level was set at 0.05, and all tests were two sided.

## 3. Results

### 3.1. Clinical Characteristics of Patients in Derivation and Validation Cohort

The clinical characteristics of HCC patients after hepatectomy in derivation cohort (*n* = 3400) and validation cohort (*n* = 1456) are listed in Table 1. Total 13 categorical variables were selected to reflect these patients' demographics and clinicopathologic conditions. And no significant differences were found among these variables between the derivation cohort and validation cohort (*P* > 0.05 in all cases).

### 3.2. Evaluation of Three AJCC Editions

**Figure 1:**
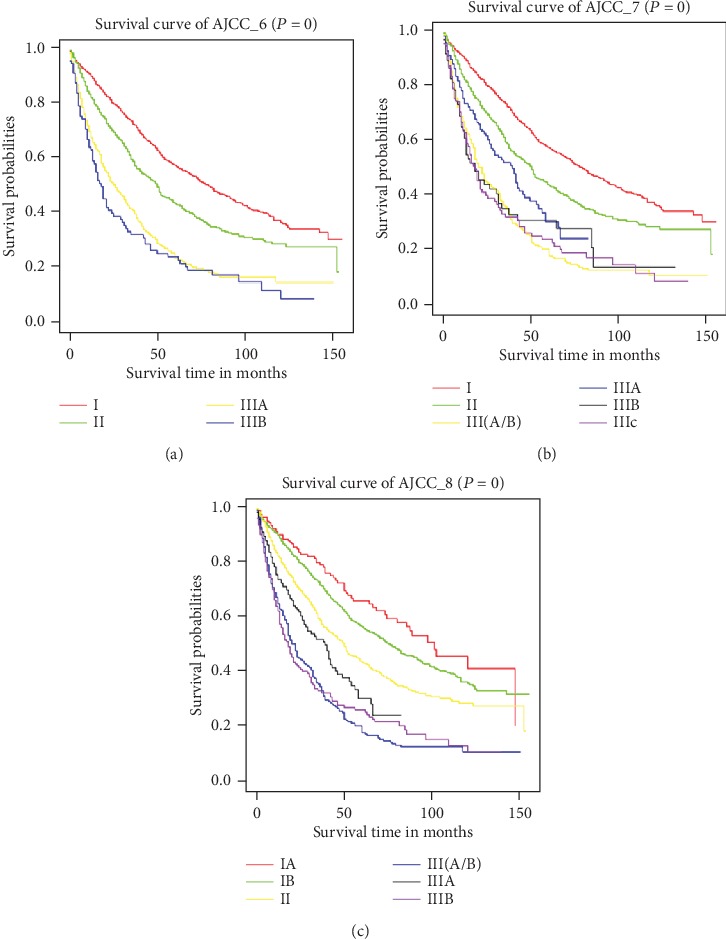
Kaplan-Meier survival curves of the derivation cohorts generated for different American Joint Committee on Cancer (AJCC) editions. (a–c) AJCC sixth, seventh, and eighth edition, respectively.

In order to compare the accuracy of three AJCC editions (6/7/8) for predicting the prognosis of HCC patients after hepatectomy, Kaplan-Meier curves were formed for each of the staging edition. The results are shown in Figure 1. Although all the curves displayed clear prognostic stratification, some overlapping was observed between the survival curves of stages IIIA and IIIB in AJCC sixth edition (Figure 1(a)), the same as stages IIIA, IIIB, and IIIC in the seventh edition (Figure 1(b)). While no overlapping was observed in the eighth edition (Figure 1(c)), except for the survival curve of III(A/B). Considering stage III(A/B) itself could not make clear staging differences from IIIA and IIIB, the AJCC eighth edition displayed higher accuracy than the other two editions by clearly distinguishing the prognosis of HCC patients with different stages.

### 3.3. Prognostic Nomogram for Overall Survival (OS)

**Figure 2:**
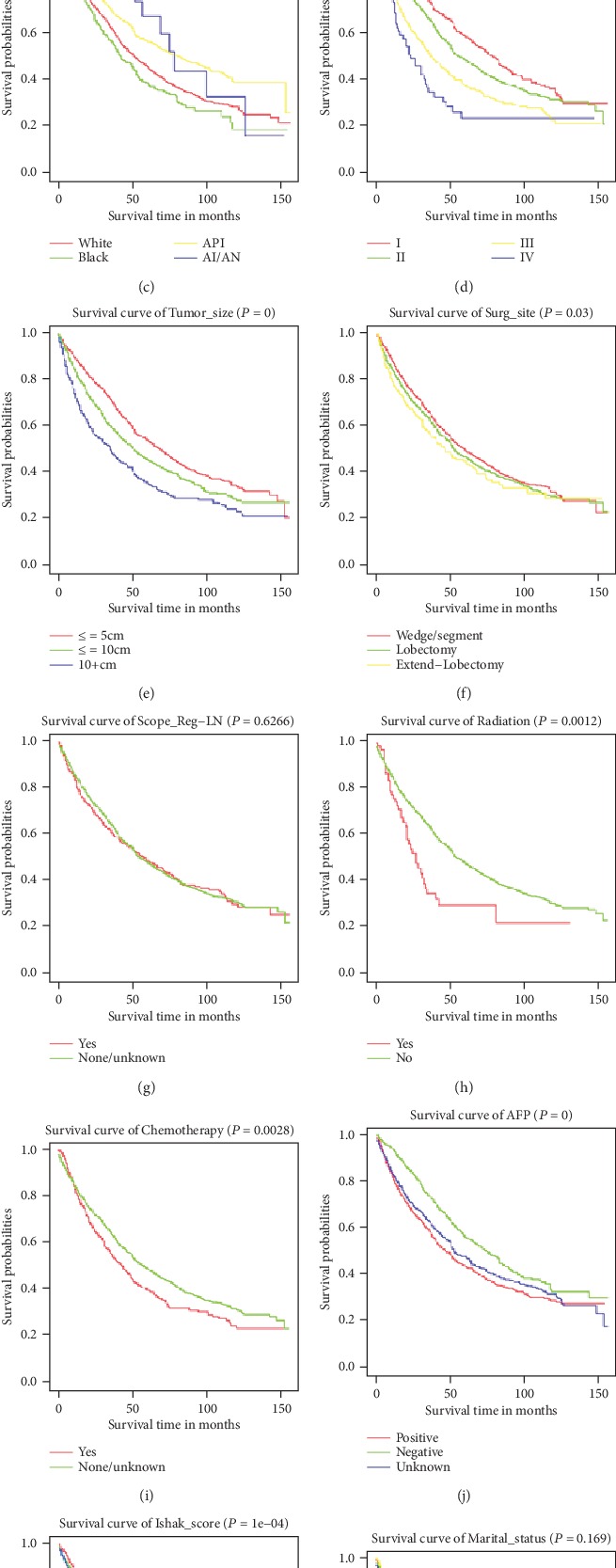
Kaplan-Meier survival curves of the derivation cohorts generated for relative risk factors. (a–l) Possible correlations between Survival and 13 risk factors (Age, Sex, Race, Grade, AJCC_8, Tumor_size, Surg_size, Scope_Reg-LN, Radiation, Chemotherapy, AFP, Ishak_score, and Marital_status). All using univariate Cox analysis.

**Figure 3:**
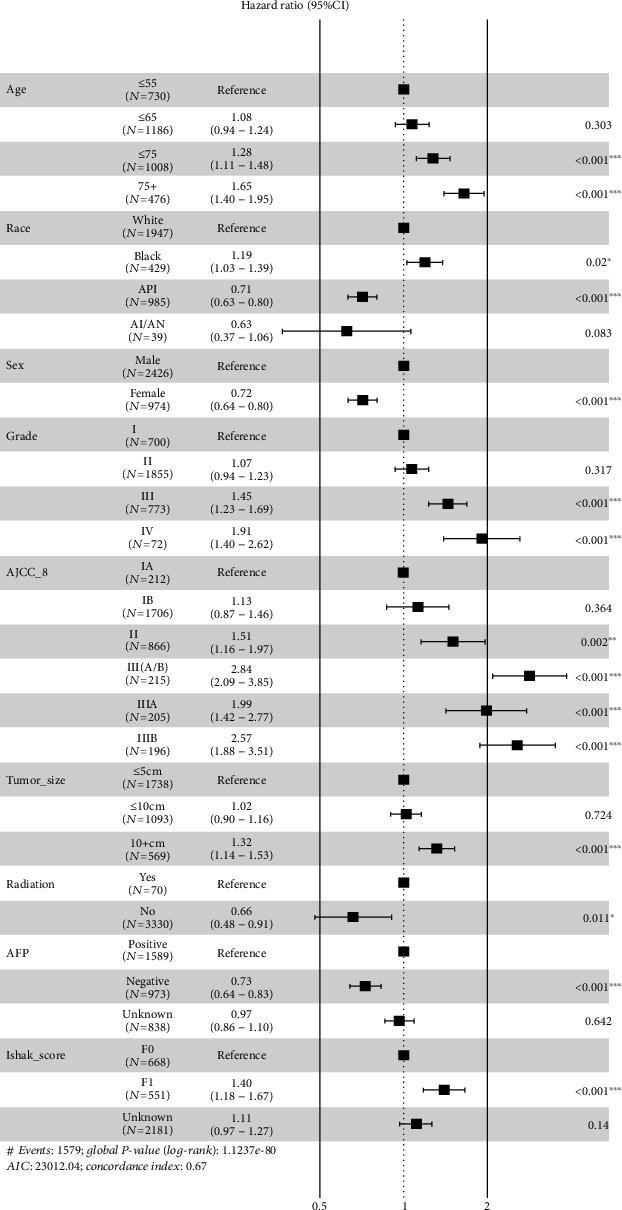
Multivariate analysis of the derivation cohort.

The correlations between survival and 13 categorical variables were evaluated by univariate Cox analysis for the patients in the derivation cohort. The results are listed in Figure 2. In which variables with a significant *P* value (*P* < 0.05) were further enrolled in multivariate Cox regression to identify the potential independent risk factors of overall survival. The results of multivariate analyses of survival are listed in Figure 3, which identified Age, Race, Sex, Grade, AJCC_8, Tumor_size, Radiation, level of *α*-fetoprotein prior to treatment (AFP), and Ishak_score as independent risk factors of OS.

**Figure 4:**
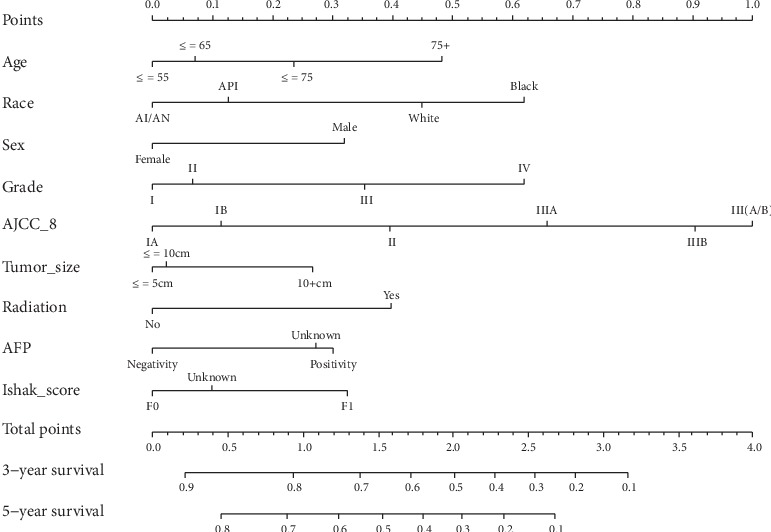
Nomogram predicting 3-/5-year survival probability of HCC patients after hepatectomy. Total points are gained by adding up all the points of each variable, and the vertical projections on lower axis present the 3-/5-year survival probability of HCC patients after hepatectomy. AFP: the highest serum *α*-fetoprotein level prior to treatment.

**Figure 5:**
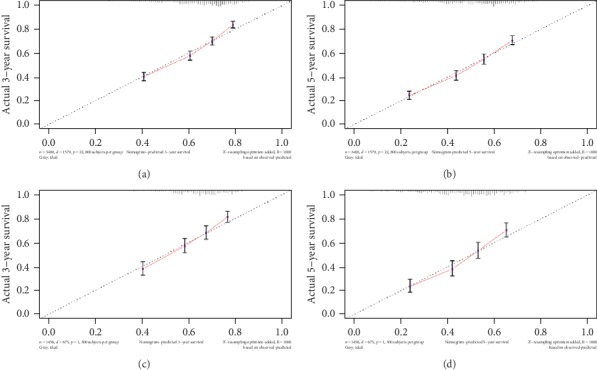
Calibration plot of the nomogram in the derivation cohort and validation cohort. The *x*-axis is the predicted survival calculated by the nomogram, and the *y*-axis is the actual survival estimated by the Kaplan-Meier method.

The prognostic nomogram was established by integrating all the nine independent factors closely related to OS in the derivation cohort (Figure 4). The C-index of the established nomogram for OS prediction was 0.667 (95% CI, 0.653 to 0.681) and was confirmed to be 0.663 (95% CI, 0.640 to 0.686) through validation cohort verification, which suggested good discrimination of our model. The calibration plot showed a good agreement between the actual outcomes and nomogram prediction in the probability of 3-/5-year survival (Figures 5(a) and 5(b)). And the calibration plot of the nomogram in the validation cohort is similar to the derivation cohort, which suggests that the nomogram based on derivation cohort is valid and stable (Figures 5(c) and 5(d)).

### 3.4. Comparison of Accuracy for OS Prediction between Nomogram and AJCC Staging System

It has been proved that AJCC eighth edition has significant advantages over other AJCC editions for predicting overall survival. Therefore, for comparison, the C-index of the AJCC8 staging system for survival prediction was 0.613 (95% CI, 0.597 to 0.629), both of the C-index for the nomogram in derivation cohort (0.667, 95% CI, 0.653 to 0.681) and validation Cohort (0.663, 95% CI, 0.640 to 0.686) were significantly higher than that of the AJCC8 (*P* < 0.05).

### 3.5. Risk Stratification of the Nomogram

**Figure 6:**
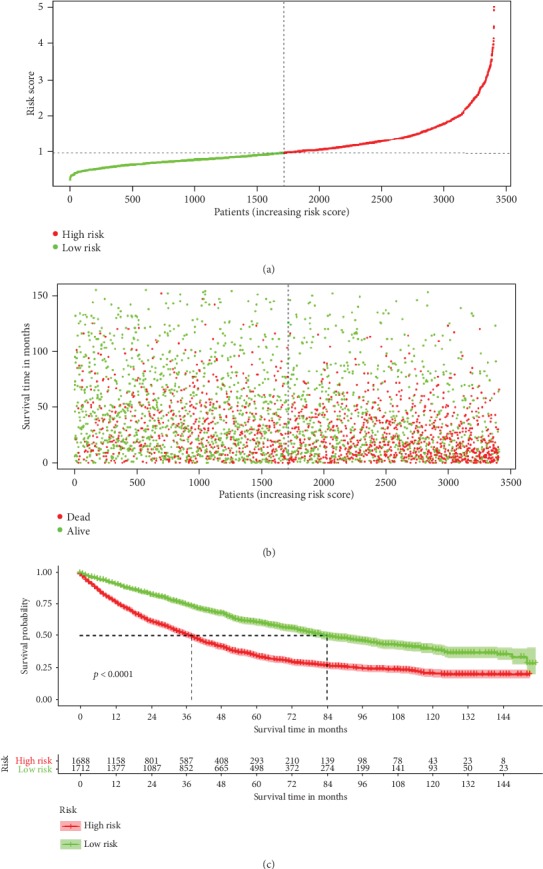
Kaplan-Meier survival curves of HCC patients with high risk scores versus low risk scores.

To determine the performance of the established nomogram in stratifying risk of HCC patients, we defined those with a risk score higher than the median in the derivation cohorts as high risk, otherwise, defined as low risk (Figure 6(a)). The survival curves generated according to the nomogram-based high and low risk stratification were shown in Figure 6(c). The survival times were significantly differentiated between these two subgroups (Figures 6(b) and 6(c), *P* = 0).

## 4. Discussion

Our study proposed a risk prediction model for predicting OS of HCC patients after hepatic resection. After certification, the C-index indicates that the prognostic value of this current nomogram was superior to AJCC staging system; it provides patients and health workers with a more convenient and friendly method to predict postoperative life expectancy. The nomogram integrated nine independent risk factors of OS of these patients to generate a total risk score that could be converted into 3-/5-year postoperative survival probability.

Based on this model, the older, black, and male patients share the higher risk of shorter survival. To the best of our knowledge, the advanced tumor grade is associated with a poor overall survival [9]. We analyzed the potential risk of advanced tumor grade; it turns out that compared with grade 1, advanced grades had much higher risk of poor prognosis (HR: 1.45 [CI: 1.23-1.69] for grade 3; HR: 1.91 [CI: 1.40-2.62] for grade 4). Tumor size is considered to be an important prognostic indicator of overall survival of postoperative HCC patients, also recognized as one of the main factors influencing the prognostic management of HCC [10, 11]. There is nearly no doubt that the outcomes are much better for small tumor (≤5 cm) [12]; however, some studies have also shown that the prognosis of excising larger tumors (>5 cm) is also promising [13]. In this study, tumor size was verified to be an independent risk factor for OS, with a HR of 1.32 (CI: 1.14-1.53) with a size over 10 cm, compared with those smaller than 5 cm.

Radiation therapy plays an important role in relieving tumor burden and delaying the progression of HCC, especially for patients who are no longer suitable for surgery. However, the limitation of radiotherapy is also explicit; since both the tumor tissue and adjacent normal liver tissue are sensitive to radiation, it is inevitable to cause some unexpected damage [14]. Recent study found that proton beam therapy (PBT) can make compensation to this unbiased damaging process. PBT can not only reduce radiation-related hepatotoxicity but also enhance sensitivity of tumor tissue to radiation dose [15]. According to this study, HCC patients who underwent radiation therapy had higher risk of suffering poorer OS, with a HR of 1.51 (CI: 1.10-2.08), which suggests traditional radiation therapy as an independent adverse prognostic factor of OS.

*α*-Fetoprotein (AFP) is often reported to be elevated in HCC patients accompanied with large tumor, early tumor recurrence, and vascular invasion [16]. Previous studies have shown that preoperative or postoperative AFP levels were highly correlated with the prognosis of HCC after hepatic resection. HCC patients with preoperative serum AFP ≤ 20 ng/mL and without surgical contraindications predict better prognosis after surgical treatment (compared with AFP > 20 ng/mL) [17]. Silva et al. underlined the importance of baseline AFP levels in HCC: as baseline AFP levels increase in unadjusted populations, the median overall survival of HCC patients dramatically decreases. And considering AFP as a continuous variable rather than a categorical one helps to further understand the correlation between baseline AFP levels and overall survival of HCC patients [18]. In our study, we verified AFP as an independent risk factor of postoperative survival of HCC patients (HR: 1.37, CI: 1.20-1.56). Considering the inconsistency of studies about AFP as a useful prognostic factor, for example, Giannini et al. found that AFP did not play an important role in predicting the prognosis of small hepatocellular carcinoma [19]. Therefore, AFP just took up a small proportion in the total points of nomogram. Further studies are urgently needed to explore the role of AFP in HCC prognosis.

In this study, calibration plots showed consistency between the predict and actual survival, which ensured the accuracy and reliability of the prognostic nomogram. According to the nomogram, HCC patients can be divided into high-risk and low-risk subgroups, which indicate significantly different overall survival rates. The main benefits of this study lies in the following aspects. First, the nomogram is based on factors available in the patient's preoperative assessment and can assists with the planning of individual postoperative surveillance protocols of HCC patients. Second, SEER database is a large public database which can provide amounts of case samples. This SEER-based study belongs to a real-world research. Third, this nomogram integrates several prognosis-related clinicopathologic factors and have been verified to work more effectively than AJCC staging system alone, which can provide a doctor-patient friendly and effective screening tool for assessing the prognosis of HCC with hepatic resection. Nevertheless, this study also has limitations, with SEER database mainly based on American population, to some extent, regional and racial differences might affect the final outcomes. And there are other factors that may also affect HCC prognosis, which are not included in this current study. In the next step, we need to find out and put more available and potential factors into further research.

## Figures and Tables

**Table 1:** Demographics and clinicopathologic characteristics of patients with HCC (Abbreviations: Surg_site: surgery of primary site; AFP: the highest serum *α*-fetoprotein level prior to treatment; Scope_Reg-LN: scope of regional lymph node surgery; Marital_status: marital status at diagnosis; API: Asian or Pacific Islander; AI/AN: American Indian/Alaska Native).

Demographic character	Derivation cohort (*n* = 3400)		Validation cohort (*n* = 1456)		*P*
	No. of patients	%	No. of patients	%	
Age					0.158
≤55	730	21.5	311	21.4	
≤65	1186	34.9	467	32.1	
≤75	1008	29.6	474	32.6	
75+	476	14.0	204	14.0	
Race					0.500
White	1947	57.3	831	57.1	
Black	429	12.6	182	12.5	
API	985	29.0	433	29.7	
AI/AN	39	1.1	10	0.7	
Sex					
Male	2426	71.4	1034	71.0	
Female	974	28.6	422	29.0	
Grade					0.832
I	700	20.6	309	21.2	
II	1855	54.6	802	55.1	
III	773	22.7	314	21.6	
IV	72	2.1	31	2.1	
AJCC_8					0.803
IA	212	6.2	93	6.4	
IB	1706	50.2	738	50.7	
II	866	25.5	359	24.7	
III(A/B)	215	6.3	80	5.5	
IIIA	205	6.0	95	6.5	
IIIB	196	5.8	91	6.3	
Tumor_size					0.328
≤5 cm	1738	51.1	778	53.4	
≤10 cm	1093	32.1	443	30.4	
10+ cm	569	16.7	235	16.1	
Surg_site					0.155
Wedge/segment	1936	56.9	849	58.3	
Lobectomy	1170	34.4	505	34.7	
Extend-lobectomy	294	8.6	102	7.0	
Scope_Reg-LN		0.0			0.343
Yes	464	13.6	184	12.6	
None/unknown	2936	86.4	1272	87.4	
Radiation					0.757
Yes	70	2.1	32	2.2	
No	3330	97.9	1424	97.8	
Chemotherapy					0.479
Yes	436	12.8	176	12.1	
No/unknown	2964	87.2	1280	87.9	
AFP					0.318
Positive	1589	46.7	676	46.4	
Negative	973	28.6	444	30.5	
Unknown	838	24.6	336	23.1	
Ishak_score					0.713
F0	668	19.6	301	20.7	
F1	551	16.2	234	16.1	
Unknown	2181	64.1	921	63.3	
Marital_status					0.434
Married	2079	61.1	898	61.7	
Never	535	15.7	248	17.0	
Ever	661	19.4	258	17.7	
Unknown	125	3.7	52	3.6	

## Data Availability

All data or model used during the study are available from the corresponding author by request.
